# The Effect of Schwann Cells/Schwann Cell-Like Cells on Cell Therapy for Peripheral Neuropathy

**DOI:** 10.3389/fncel.2022.836931

**Published:** 2022-03-08

**Authors:** Qian Wang, Fang-Yu Chen, Zhuo-Min Ling, Wen-Feng Su, Ya-Yu Zhao, Gang Chen, Zhong-Ya Wei

**Affiliations:** ^1^Key Laboratory of Neuroregeneration of Jiangsu and Ministry of Education, Jiangsu Clinical Medicine Center of Tissue Engineering and Nerve Injury Repair, Co-innovation Center of Neuroregeneration, Nantong University, Nantong, China; ^2^Medical School of Nantong University, Nantong, China; ^3^Department of Anesthesiology, Affiliated Hospital of Nantong University, Nantong, China

**Keywords:** Schwann cells, Schwann cell-like cells, myelination, regeneration, peripheral neuropathy

## Abstract

Peripheral neuropathy is a common neurological issue that leads to sensory and motor disorders. Over time, the treatment for peripheral neuropathy has primarily focused on medications for specific symptoms and surgical techniques. Despite the different advantages of these treatments, functional recovery remains less than ideal. Schwann cells, as the primary glial cells in the peripheral nervous system, play crucial roles in physiological and pathological conditions by maintaining nerve structure and functions and secreting various signaling molecules and neurotrophic factors to support both axonal growth and myelination. In addition, stem cells, including mesenchymal stromal cells, skin precursor cells and neural stem cells, have the potential to differentiate into Schwann-like cells to perform similar functions as Schwann cells. Therefore, accumulating evidence indicates that Schwann cell transplantation plays a crucial role in the resolution of peripheral neuropathy. In this review, we summarize the literature regarding the use of Schwann cell/Schwann cell-like cell transplantation for different peripheral neuropathies and the potential role of promoting nerve repair and functional recovery. Finally, we discuss the limitations and challenges of Schwann cell/Schwann cell-like cell transplantation in future clinical applications. Together, these studies provide insights into the effect of Schwann cells/Schwann cell-like cells on cell therapy and uncover prospective therapeutic strategies for peripheral neuropathy.

## Introduction

Peripheral neuropathies are commonly encountered disorders that result from a great number of etiologies, including trauma and side effects of diseases and treatments (Hughes, 2002). Although there is no standard method to diagnose peripheral neuropathy, the development of imaging and laboratory tests has aided in primary diagnosis, and electromyography and nerve conduction tests are especially beneficial for allowing doctors to narrow down the category and the management of peripheral neuropathies (Barrell and Smith, 2019). The categories used to be mononeuropathies, multifocal neuropathies and polyneuropathies. However, these categories are frequently further divided into axonal, demyelinating, or mixed according to a systematic approach, which is vital for treatment (Hanewinckel et al., 2016). The symptoms often include sensory and motor dysfunctions, including numbness, pain, weakness and paresthesia due to damage to sensory, motor and autonomic fibers. Treatments for peripheral neuropathy are primarily dependent on the subtype and cause of underlying disease, such as grafts for traumatic nerve injury (Baradaran et al., 2021) and metabolic control for diabetic neuropathy (Cernea and Raz, 2021; Holmes and Hastings, 2021). Recently, with insights into cell-based therapy for diseases, emerging evidence has revealed the benefits of cell transplantation in peripheral neuropathic conditions (Hopf et al., 2020; Monje, 2020).

**FIGURE 1:**
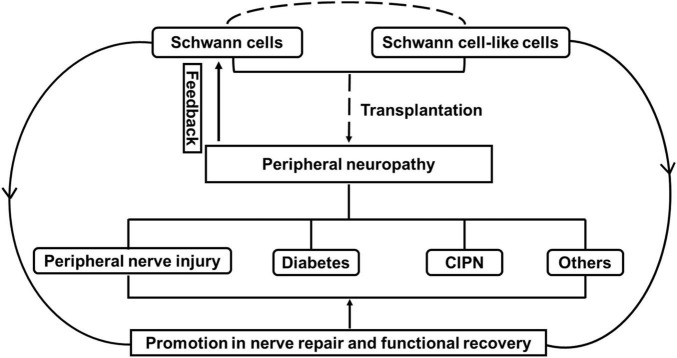
The effect of Schwann cells and Schwann cell-like cells on cell therapy for peripheral neuropathy. Note that peripheral neuropathies induced by peripheral nerve injury, diabetes and chemotherapy-induced peripheral neuropathy (CIPN) often leads to the malfunctional change in Schwann cells. Transplantation with Schwann cells or Schwann cell-like cells (from different sources) attempts to promote nerve repair and functional recovery through the effect of Schwann cells for the treatment of peripheral neuropathies.

Peripheral neuropathies are affected by disorders of peripheral nerve fibers and cells (Hughes, 2002; Hanewinckel et al., 2016; Barrell and Smith, 2019; Hammi and Yeung, 2021). Schwann cells, which are the primary glial cells in the peripheral nervous system, are predominantly subdivided into myelinating and non-myelinating Schwann cells, both of which are associated with axons through physical support and the release of a variety of neurotrophins and many other signaling molecules during development (Kidd et al., 2013). Relatively large-diameter axons from most motor axons, some sensory axons and are enwrapped by Schwann cells, resulting in the establishment of compact myelin at a ratio of 1:1, which is needed for fast nerve conduction. Other small-diameter axons from autonomous and many sensory neurons, which are known as Remak bundles, are wrapped only by Schwann cells and are not myelinated (Griffin and Thompson, 2008). Schwann cells are recognized as flexible cells due to their capability for rapid transformation after injury (Jessen and Mirsky, 2016). In the injured microenvironment, myelinating Schwann cells and non-myelinating Remak Schwann cells coordinate to repair Schwann cells, resembling the developmental stage through the self-renewal and release of a variety of neurotrophic factors and signaling molecules involved in motor and sensory functional recovery (Stassart and Woodhoo, 2021). Therefore, the role of Schwann cells is pivotal for axonal functions both in physiological and pathological conditions, which leads to increasing attempts to prevent malfunction in Schwann cells or the supply Schwann cells/Schwann cell-like cells for the treatment of peripheral neuropathies (Brewer et al., 2016; Sayad Fathi and Zaminy, 2017; Wing et al., 2017; Al-Massri et al., 2020; Hopf et al., 2020; Monje, 2020). Schwann cells are known to originate from neural crest cells, which can be found in other tissues, such as the epidermis and hair follicle, and have great potential to generate Schwann cell-like cells (McKenzie et al., 2006; Lin et al., 2011). Moreover, with technical innovations in related stem cells, many types of stem cells can differentiate into Schwann cell-like cells or target the regulation of Schwann cells for motor and sensory functional recovery (Caddick et al., 2006; Park et al., 2010; Ma et al., 2015; Cai et al., 2017; Hopf et al., 2020). Thus, in this review, we will primarily discuss the potential applications of Schwann cells/Schwann cell-like cells in peripheral neuropathies induced by common disorders, including peripheral nerve injury, diabetes and chemotherapy, and the challenges for future clinical treatments (Figure 1).

## Peripheral Nerve Injury-Induced Neuropathy

Peripheral nerve injury is a common disease that results from trauma or disease and leads to damage to motor and sensor functions. Although the peripheral nervous system has the potential to self-repair nerve injury, peripheral nerve injury-induced neuropathy and lifelong disabilities for patients are common (Menorca et al., 2013). Insights into cellular and molecular mechanisms have revealed that modulating axons and Schwann cells are effective strategies for peripheral nerve injury-induced neuropathy. After injury, injured axons break and form debris in the distal stump, which is called Wallerian degeneration. This debris is segmented and incorporated by Schwann cells, and then phagocytized with the aid with the recruited macrophages (Nazareth et al., 2021). Once this debris is cleaned, the proximal stump will begin to outgrow. During this process, Schwann cells play an important role in the repair of peripheral nerve injury-induced neuropathy. Once axonal injury occurs, activated Schwann cells transform into a dedifferentiated state by expressing developmental genes, releasing various neurotrophic factors to create a reparative environment, and forming Büngner bands, which are a longitudinal column for guiding axonal regrowth through proliferation in the distal stump (Stassart and Woodhoo, 2021). However, this self-repair method is unable to guide axonal outgrowth and target innerved muscles due to a lack of an advantageous environment, which includes the dysfunction of Schwann cells (Lehmann and Hoke, 2016). Therefore, emerging evidence is focused on cell transplantation to supply Schwann cells or repair Schwann cells to promote axonal growth and motor and sensory restoration (Lehmann and Hoke, 2016; Hopf et al., 2020). Among these strategies, cell transplantation in combination with nerve scaffolds is a promising treatment for peripheral nerve injury-induced neuropathy (Rodriguez et al., 2000; Kornfeld et al., 2019). In the case of peripheral nerve injury, the gold standard treatment is end-to-end suturing of the proximal and distal parts by neurosurgical methods. However, this method is only useful for short gaps (<3 mm), and for longer gaps, a nerve or conduit graft is required to bridge the gap (Hopf et al., 2020). Thus, autologous Schwann cell transplantation is the best choice for treatment. However, these cells must be collected from healthy peripheral nerves and harvested in a time-consuming manner, and all of these limitations constrain their wide applications (Sullivan et al., 2016; Baradaran et al., 2021). Therefore, attention has moved toward the use of allogeneic Schwann cells and Schwann cell-like cells from stem cells to promote axonal regeneration and repair peripheral nerve injury-induced neuropathy (Sayad Fathi and Zaminy, 2017; Hopf et al., 2020; Kubiak et al., 2020). Here, we review current developments in Schwann cell or Schwann cell-like cell transplantations for the repair of peripheral nerve injury-induced neuropathy (Tables 1, 2).

**TABLE 1:** The effect of Schwann cell therapy on peripheral nerve injury-induced neuropathy.

Model	Schwann cell source	Outcomes	Notes
Rat sciatic nerve defect with an 8 mm gap	Autologous	Extensive peripheral nerve regeneration and myelination	• A strong immune reaction occurred when seeding with heterologous Schwann cells; • Seeding density of Schwann cells should be considered (Guenard et al., 1992)
Sciatic nerve defect with a 5 mm gap in immune-deficient rats	Allogeneic, from human nerves	Promotion of axonal regeneration and myelination	Repair outcomes were better than the channels with Matrigel solution alone (Levi et al., 1994)
Human sciatic nerve defect with a 7.5 cm gap	Autologous	Proximal sensory recovery, including neuropathic pain, and motor function recovery in the common peroneal and tibial distribution	The patient suffered complete transection of sciatic nerves by a boat propeller injury (Levi et al., 2016)
Human sciatic nerve defect with a 5 cm gap	Autologous	Recovery of complete motor function and partial sensation in the tibial distribution	The patient suffered partial damage of the tibial division of sciatic nerves by a gun wound to the leg (Gersey et al., 2017)
Mouse sciatic nerve crush	Allogeneic, from human skin	Promotion of axonal regrowth and myelination	• Adult human skin-derived Schwann cells were similar to human nerve-derived Schwann cells in genetical and phenotypical characterization; • Highly accessible source of autologous skin-derived Schwann cells was a substitute for nerve-derived Schwann cells for injured nerve repair (Stratton et al., 2017)
Rat sciatic nerve defect with a 10 mm gap	Allogeneic, from neonatal rat sciatic nerves	Improvement in axonal regeneration	• The quantity of regenerated axons was less than that induced by treatment with syngeneic Schwann cells; • Immune response occurred at 6 weeks post-transplantation in the absence of immunosuppressive therapy (Mosahebi et al., 2002)
Rat sciatic nerve defect with a 20 mm gap (Hoben et al., 2015), 10 mm gap (Sun et al., 2009) and 14 mm gap (Santosa et al., 2013)	Allogeneic, from neonatal (Sun et al., 2009; Hoben et al., 2015)/adult (Santosa et al., 2013) rat sciatic nerves	Improvement in axonal regeneration (Sun et al., 2009; Santosa et al., 2013; Hoben et al., 2015) and myelination (Sun et al., 2009)	• Acellular nerve allografts combined with allogeneic Schwann cells obtained the same outcomes as the isograft group (Hoben et al., 2015); • Adding vascular endothelial growth factor alone (Hoben et al., 2015) or Schwann cells overexpressing glial cell-derived neurotrophic factor (Santosa et al., 2013) in acellular nerve allografts had reduced effects on improving axonal regeneration
Rat sciatic nerve injury with a 3 cm gap	Autologous, from the proximal stump neuroma	Regenerative fibers crossing the entire distance but no motor and poor sensory function recovery	It is challenging to regenerate axons with a 3 cm gap defect with only grafts (Aszmann et al., 2008)
Primate ulnar nerve defect with a 6 cm gap	Autologous, from the sural nerve fascicles	Low immune response and significant regeneration	Cold-preserved allografts combined with autologous Schwann cells was a potentially safe and effective alternative to autografts (Hess et al., 2007)
Rabbit peroneal nerve defect with a 6 cm gap	Autologous, from the contralateral peroneal nerve	Excellent growth of axons targeting the distal end	Autologous Schwann cells break the limit of nerve regeneration by an empty autogenous venous nerve conduit (Strauch et al., 2001)
Rat sciatic nerve defect with a 10 mm gap (Mosahebi et al., 2002) and 1 cm gap (Bryan et al., 2000; Tohill et al., 2004; di Summa et al., 2011)	Allogeneic, from rat sciatic nerves	Improvements in axonal regrowth and fiber myelination	Combination with allogeneic Schwann cells obtained better outcomes in synthetic grafts, such as polyhydroxybutyrate conduits (Mosahebi et al., 2002; Tohill et al., 2004), fibrin conduits (di Summa et al., 2011) and poly (lactic-co-glycolic) acid conduits (Bryan et al., 2000)

**TABLE 2:** The effect of Schwann cell-like cells on cell therapy for peripheral nerve injury-induced neuropathy.

Model	Cell source	Grafts	Outcomes	Notes
Rat sciatic nerve transection with a 12 mm gap (Mimura et al., 2004; Ao et al., 2011), 10 mm gap (Shimizu et al., 2007); Rabbit facial nerve buccal branch transection with a 1 cm gap (Wang et al., 2011)	BMSC-derived Schwann cells from rats (Mimura et al., 2004; Ao et al., 2011), humans (Shimizu et al., 2007), rabbits (Wang et al., 2011)	Hollow fiber (Mimura et al., 2004) Transpermeable tube (Shimizu et al., 2007) Chitosan nerve conduits (Ao et al., 2011), autogenous vein (Wang et al., 2011)	Improvements in regenerative axon populations (Mimura et al., 2004; Shimizu et al., 2007; Ao et al., 2011; Wang et al., 2011), motor functions and reconstruction of Ranvier nodes and myelination (Mimura et al., 2004; Ao et al., 2011; Wang et al., 2011)	• No tumor formation within 6 months (Mimura et al., 2004) • Human BMSCs were used in rat sciatic nerve repair with immunosuppressants (Shimizu et al., 2007) • No significant outcomes compared with sciatic nerve-derived Schwann cells (Ao et al., 2011)
Rat sciatic nerve transection with a 1 cm gap (di Summa et al., 2010, 2011; Orbay et al., 2012; Kingham et al., 2014) and 15 mm gap (Georgiou et al., 2015); Tibial nerve crush in athymic nude rats (Tomita et al., 2013)	ASC-derived Schwann cell-like cells from rats (di Summa et al., 2010, 2011; Orbay et al., 2012; Georgiou et al., 2015) and humans (Tomita et al., 2013; Kingham et al., 2014)	Nerve fibrin conduits (di Summa et al., 2010, 2011; Kingham et al., 2014) Silicone (Orbay et al., 2012) Aligned collagen matrix (Georgiou et al., 2015)	Improvements in axonal regeneration (di Summa et al., 2010, 2011; Orbay et al., 2012; Tomita et al., 2013; Kingham et al., 2014; Georgiou et al., 2015) and myelination (Orbay et al., 2012; Tomita et al., 2013), as well as a reduction in muscle atrophy (di Summa et al., 2011); neurotrophic factor release (Kingham et al., 2014), and glial cell differentiation (Tomita et al., 2013); an increase in conduit vascularity (Kingham et al., 2014)	• Differentiated ASC transplantation obtained similar outcomes as that with differentiated MSCs at 2 weeks (di Summa et al., 2010), but the treatment was more effective than differentiated MSCs in a long-term experiment of 16 weeks (di Summa et al., 2011); • Differentiated and undifferentiated rat ASCs had a similar effect on nerve reconstruction 6 months after transplantation (Orbay et al., 2012); • Differentiated human ASCs had a potent effect on neurotrophic factors release and axonal regeneration (Tomita et al., 2013)
Rat sciatic nerve transection with an 8 mm gap (Matsuse et al., 2010)	Schwann cells differentiated from human umbilical cord-derived MSCs	Matrigel-transplanted graft	Promotion of nerve regeneration and myelination	• FK506 was used to avoid immunorejection; • The effect was comparable to treatment with human Schwann cells
Mouse sciatic nerve crush (McKenzie et al., 2006); Rat sciatic nerve crush (Kumar et al., 2016; Stratton et al., 2016; Wu et al., 2020); Rat sciatic nerve transection with a 5 mm gap (Shakhbazau et al., 2014; Zhang et al., 2014), 10 mm gap (Khuong et al., 2014; Zhu et al., 2018)	SKP-derived precursor Schwann cells from mice (McKenzie et al., 2006) and rats (Khuong et al., 2014; Shakhbazau et al., 2014; Zhang et al., 2014; Kumar et al., 2016; Stratton et al., 2016; Zhu et al., 2018; Wu et al., 2020; Cong et al., 2021)	Silicon tube (Shakhbazau et al., 2014) Decellularized nerve grafts (Khuong et al., 2014) Artificial guidance channels (Zhang et al., 2014) Chitosan nerve guidance conduits and silk fibroin filamentous fillers (Zhu et al., 2018)	Improvements in axonal regeneration (McKenzie et al., 2006; Zhang et al., 2014), and myelination (Kumar et al., 2016; Zhu et al., 2018), sensory functional recovery (Shakhbazau et al., 2014), motoneuron and sensory neuron regrowth (Wu et al., 2020; Cong et al., 2021), behavioral recovery (Khuong et al., 2014), surrounding immunological properties to accelerate myelin debris clearance (Stratton et al., 2016)	• The probability of myelination with SKP-derived Schwann cells was higher than with naïve SKPs at 2 weeks post-transplantation, but they had similar profiles at 4 weeks (McKenzie et al., 2006); • Cells in supporting sensory functional recovery is similar to treatment with isogenic Schwann cells (Shakhbazau et al., 2014); • Cells improved behavioral recovery in both acute and chronic nerve injury, but the medium and the dead cells had fewer effects (Khuong et al., 2014); • The immunomodulatory role of SKP- derived precursor Schwann cells on peripheral neuropathy included macrophage recruitment and inflammatory factor expression (Stratton et al., 2016); • Transplantation with adult SKP-derived Schwann cells produced the same outcome that of acutely injured Schwann cells, but chronically denervated Schwann cells were less effective (Kumar et al., 2016); • Acellular matrix from SKP-derived Schwann cells combined with chitosan/silk scaffolds was beneficial for nerve repair (Zhu et al., 2018);
				• Extracellular vesicles from SKP-derived Schwann cells were responsible for axonal regrowth of motoneurons and sensory neurons (Wu et al., 2020; Cong et al., 2021)
Mouse sciatic nerve transection with a 2–3 mm gap (Kim et al., 2017)	Schwann cell-like cells from human pluripotent stem cells	Matrigel	Improvements in nerve regeneration and myelination	Cells derived from human pluripotent stem cells *via* self-renewing Schwann cell precursors under sequential treatments with cultured medium
Mouse sciatic nerve transection with a 5 mm gap (Sowa et al., 2017)	Schwann cell-like cells from direct conversion from human fibroblast	Gelatin hydrogel	Improvements in myelin formation, axonal regrowth and motor functional recovery	The effect of cells in axonal regrowth and motor functional recovery was comparable to that of treatment with Schwann cells from peripheral nerves
Rat sciatic nerve transection with a 15 mm or 12 mm (Verdú et al., 1999; You et al., 2011; Zhang et al., 2019), and 20 mm gap (Guérout et al., 2011; Boecker et al., 2018); Rat facial nerve transection with a 5 mm gap (Guntinas-Lichius et al., 2001) or end-to-end suture (Guntinas-Lichius et al., 2002); Rat sciatic nerve transection with microsurgical nerve repair (Radtke et al., 2009a); Rat sciatic nerve crush lesion (Dombrowski et al., 2006); Mouse sciatic nerve transection with a 3 mm gap (Goulart et al., 2016)	Olfactory bulb ensheathing cells (Verdú et al., 1999; Guntinas-Lichius et al., 2001; Dombrowski et al., 2006; Radtke et al., 2009a; You et al., 2011; Goulart et al., 2016; Boecker et al., 2018; Zhang et al., 2019); Olfactory mucosa (Guntinas-Lichius et al., 2002)	Silicone tube prefilled with a laminin gel (Verdú et al., 1999); Microporous poly acid conduit (You et al., 2011); Tubular conduit (Goulart et al., 2016); Nerve guide Perimaix (Boecker et al., 2018); Nerve conduits (Zhang et al., 2019)	Improvements in regenerative axon populations (Verdú et al., 1999); Stimulation on collateral sprouting (Guntinas-Lichius et al., 2001); Promotion in the accuracy of target reinnervation and the vibrissae motor performance (Guntinas-Lichius et al., 2002); Improvements in axonal regeneration and functional outcomes (Radtke et al., 2009a; Guérout et al., 2011); Improvements in myelination and nodal formation of regenerative peripheral nerve fibers (Dombrowski et al., 2006); Synergistical improvements in Schwann cells-mediated sciatic nerve repair (You et al., 2011); Improvements in sciatic nerve functional and morphological recovery (Goulart et al., 2016; Boecker et al., 2018); The increase of the level of brain derived factor and nerve growth factor (Zhang et al., 2019)	• It is a powerful tool for severe nerve injury (2 months between injury and repair) (Guntinas-Lichius et al., 2001); • Transplantation of olfactory mucosa significantly improves nerve regeneration (Guntinas-Lichius et al., 2002); • No olfactory ensheathing cells are present in the sciatic nerves 3 months post-transplantation (Guérout et al., 2011); • Olfactory ensheathing cells with the nerve guide Perimaix has local effects on nerve regeneration, but not for traversing the lesion gap (Boecker et al., 2018); • Epidermal neural crest stem cell and olfactory ensheathing cell co-transplantation effectively repairs peripheral nerve injury (Zhang et al., 2019)

### Schwann Cell Transplantation in Peripheral Nerve Injury-Induced Neuropathy

In 1992, a study of the transplantation of autologous Schwann cells derived from adult nerves in permselective guidance channels to repair 8 mm nerve gaps in transected rat sciatic nerves indicated that this combination supported extensive regeneration and myelination. In contrast, a strong immune reaction occurred when heterologous Schwann cells were seeded, resulting in the prevention of nerve regeneration (Guenard et al., 1992). To avoid immune reactions, an immune-deficient rat was used, and the functional capacity of human Schwann cells in an 8 mm gap of transected sciatic nerves was evaluated. The outcomes showed that human Schwann cells could survive and effectively promote axonal regrowth and myelination but were less successful than allogeneic Schwann cells (Levi et al., 1994). A study aimed to evaluate the effect of allogeneic Schwann cell transplantation following rat sciatic nerve injury with a 10 mm gap and showed that compared with syngenetic Schwann cells, allogeneic Schwann cells also promoted axonal regeneration and myelination, but the effect was less than that of syngenetic Schwann cells, and an immune response occurred at 6 weeks post-transplantation when there was no use of immunosuppressive therapy (Mosahebi et al., 2002). In addition, a decellularizing approach has been developed to prevent rejection when allogeneic nerve grafts are applied to injured nerve repair (Hudson et al., 2004). However, due to the loss of Schwann cells, this method is less effective for nerve repair than contact nerves (Hoben et al., 2015). Of note, decellularized nerve conduits combined with Schwann cells to repair peripheral nerve injury obtained good results in non-human primate 6 cm ulnar nerve defects (Hess et al., 2007) and rat sciatic nerve defects (Aszmann et al., 2008; Sun et al., 2009; Hoben et al., 2015) and were demonstrated to be a better therapy than the addition of vascular endothelial growth factor to improve axonal regrowth (Hoben et al., 2015). In human studies (Levi et al., 2016; Gersey et al., 2017), Schwann cells were isolated from sural nerve biopsies and traumatized sciatic nerve stumps. After purification and proliferation, the cells were combined with sural nerve grafts to repair two cases of a 7.5 cm defect (case 1 with complete transection of sciatic nerves by a boat propeller injury) and a 5 cm defect (case two with partial damage of the tibial division of sciatic nerves by a gun wound of the leg). Follow-up was 36 months for the patient in case 1, and the patient regained proximal sensory recovery, including neuropathic pain, and motor function recovery in the common peroneal and tibial distribution (Levi et al., 2016). Twelve months post-operation, the patient in case 2 exhibited recovery of complete motor function and partial sensation in the tibial distribution (Gersey et al., 2017). Despite the fact that after injury, neurotrophic factor release from activated Schwann cells is beneficial for nerve regeneration and functional recovery, a study of allogeneic nerve grafts with Schwann cells overexpressing glial cell line-derived neurotrophic factors resulted in limited axonal regeneration and poor functional recovery (Santosa et al., 2013). Indeed, the timing, volume and distribution of these neurotrophic factors associated with postinjury Schwann cell behavior are critical for the rate of axonal regrowth and functional recovery (Kidd et al., 2013; Jessen and Mirsky, 2016). Due to the difficulty of harvesting human nerve-derived Schwann cells, skin-derived Schwann cells from patients were collected, and gene expression was characterized in human nerve-derived Schwann cells, and the feasibility of transplantation into injured mouse sciatic nerves was evaluated. The results demonstrated that adult human skin-derived Schwann cells were similar to human nerve-derived Schwann cells genetically and phenotypically, which indicates that a highly accessible source of autologous skin-derived Schwann cells may be a substitute for nerve-derived Schwann cells for injured nerve repair (Stratton et al., 2017). Both autogenous and allogenous nerve transplantation require nerve supply from the donor, which leads to donor-site morbidity resulting from the loss of nerves (Kim et al., 2020). A variety of conduits have been developed, including veins and synthetic grafts. Several studies (Chiu et al., 1982; Strauch et al., 1996) have used autogenous venous nerve conduits to successfully support axonal regeneration for short distances (less than a 3 cm gap). Moreover, conduit supplementation with autologous Schwann cells rapidly grew 6 cm peroneal nerve defect-injured nerves compared with treatment alone (Strauch et al., 2001). In addition, Schwann cells in a polyhydroxybutyrate conduit (Mosahebi et al., 2002; Tohill et al., 2004), fibrin conduit (di Summa et al., 2011) and poly (lactic-co-glycolic) acid conduit (Bryan et al., 2000) display more improvements in axonal regrowth and fiber myelination than the use of conduits alone. Based on a study comparing green fluorescent protein-labeled Schwann cells with non-transduced Schwann cells in bioengineered nerve conduits, the outcomes showed that both of treatments had similar growth characteristics (Tohill et al., 2004). Fluorescently labeled Schwann cells will be beneficial for monitoring Schwann cell behaviors and interactions with axons in bioengineered systems.

### The Effect of Schwann Cell-Like Cells on Cell Therapies for Peripheral Neuropathy

Schwann cells are primary glial cells in the peripheral nervous system, and autologous and allogeneic Schwann cells are thought to be good choices for the repair of injured nerves. However, because acquiring these Schwann cells from nerves is time-consuming and has secondary morbidity at the donor site, it is desirable to explore cell sources with similar potential to produce Schwann cells. Stem cells with wide distribution, multilineage potential and self-renewal capacity are highly suitable as alternative cell sources for Schwann cells (Sayad Fathi and Zaminy, 2017). Here, we emphasize the effect of stem cell-derived Schwann cell-like cells on cell therapy for peripheral neuropathy.

A major source of Schwann cell-like cells is mesenchymal stem/stromal cells (MSCs), which are readily isolated from a variety of tissues, including bone marrow, skin, adipose tissue and umbilical cord tissue (Sayad Fathi and Zaminy, 2017; Hopf et al., 2020). Bone marrow stromal cell (BMSC)-derived Schwann cells from rats (Mimura et al., 2004; Ao et al., 2011), humans (Shimizu et al., 2007) and rabbits (Wang et al., 2011) may mediate improvements in regenerative axon populations, motor functions and the reconstruction of Ranvier nodes and myelination in rat sciatic nerve transection with a 12 mm gap and a 10 mm gap, as well as in rabbit facial nerve buccal branch transection within a 1 cm gap. Moreover, tumor formation was not detected within 6 months (Mimura et al., 2004), and no significant outcomes occurred compared with treatment with sciatic nerve-derived Schwann cells (Ao et al., 2011). Differentiated adipose-derived stem cells (ASCs) are primarily derived from rats (di Summa et al., 2010, 2011; Orbay et al., 2012; Georgiou et al., 2015) and humans (Tomita et al., 2013; Kingham et al., 2014). Transplantation with these cells in nerve fibrin conduits, silicone or aligned collagen matrix has a potential role in the repair of peripheral neuropathy to improve neurotrophic factor release, axonal regrowth (di Summa et al., 2010, 2011; Orbay et al., 2012; Tomita et al., 2013; Kingham et al., 2014; Georgiou et al., 2015), myelination (Orbay et al., 2012; Tomita et al., 2013) and vascularity (Kingham et al., 2014), as well as reduce muscle atrophy (di Summa et al., 2011). Notably, differentiated and undifferentiated rat ASCs combined with silicone in rat sciatic nerve transection with a 1 cm gap had a similar effect on nerve reconstruction within 6 months (Orbay et al., 2012). However, transplantation with differentiated human ASCs in a nude rat tibial nerve crush model obtained a better outcome than the use of undifferentiated cells (Tomita et al., 2013). In contrast to the effect of undifferentiated ASCs, differentiated ASCs had a similar effect at 2 weeks post-transplantation but were more effective in a long-term experiment of 16 weeks (di Summa et al., 2010, 2011). Although there is no direct evidence of human umbilical cord blood-MSC-derived Schwann cell-like cells for treating peripheral neuropathy (Weiss and Troyer, 2006), Schwann cell-like cells obtained from the mesenchymal tissue surrounding umbilical cord vessels (Wharton jelly) were combined with Matrigel-transplanted grafts to repair rat sciatic nerve transection with an 8 mm gap with the immunosuppressor FK506. The effect was comparable to that of using human Schwann cells to promote nerve regeneration and myelination (Matsuse et al., 2010). Compared with other stem cells, skin-derived precursor cells (SKPs) are more accessible and readily differentiate into Schwann cells, and much more attention has been given to investigating their potential role in cell therapy in peripheral nerve injury-induced neuropathy. These cells are widely used to repair rodent sciatic nerve crush or transection with 5 or 10 mm gaps combined with different kinds of grafts, including silicon tubes (Shakhbazau et al., 2014), decellularized nerve grafts (Khuong et al., 2014), artificial guidance channels (Zhang et al., 2014), and chitosan/silk scaffolds (Zhu et al., 2018). After transplantation, these cells improve sensory functional and behavioral recovery in both acute (4 weeks) and chronic (17 weeks) nerve injury (Khuong et al., 2014; Shakhbazau et al., 2014), axonal regeneration and myelination *in vivo* (McKenzie et al., 2006; Zhang et al., 2014; Kumar et al., 2016; Zhu et al., 2018), and motoneuron and sensory neuron regrowth *in vitro* (Wu et al., 2020; Cong et al., 2021). Moreover, they can adjust surrounding immunological properties to accelerate myelin debris clearance by recruiting many more macrophages and enhancing inflammatory factor expression (Stratton et al., 2016). The myelination of these cells is higher than that of naïve SKPs in the early stage (McKenzie et al., 2006), and their ability to support sensory functional recovery is equal to or better than that of treatments with isogenic Schwann cells (Shakhbazau et al., 2014). In addition to the great effect of the cells by themselves, acellular matrix and extracellular vesicles from SKP-derived cells are also responsible for neuronal regrowth *in vitro* (Wu et al., 2020; Cong et al., 2021), but dead cells or the medium was less effective on nerve repair *in vivo* (Khuong et al., 2014). Although great improvements have been obtained with rat/mouse SKP-derived Schwann cell-like cells, the clinical application of human SKPs still needs many more studies to test the utility of cells from different anatomical regions (Dai et al., 2018). In addition, human pluripotent stem cells can be differentiated into Schwann cell-like cells *via* self-renewing Schwann cell precursor cells through sequential treatment with conditioned medium *in vitro*, and the combination with Matrigel successfully improves axonal regeneration and myelin repair (Kim et al., 2017). Another interesting source of Schwann cell-like cells is human fibroblasts, which can be converted with a cellular reprogramming strategy. *In vitro* and *in vivo* experiments with gelatin hydrogel showed a potential role of converted Schwann cells in significantly enhancing axonal regrowth, myelin repair and motor functional recovery, which is comparable to treatment with Schwann cells from peripheral nerves (Sowa et al., 2017).

In addition to these Schwann cell-like cells, olfactory ensheathing cells from olfactory bulb and mucosa share many properties with Schwann cells which include the support of axonal regeneration and myelination (Doucette, 1990). They also exhibit great potentials for nerve repair in peripheral nerve injury-induced neuropathy (Radtke et al., 2009b,^2011^; Radtke and Kocsis, 2012, 2014). Olfactory bulb ensheathing cells from mouse (Goulart et al., 2016) and rat mediate improvements in axonal regeneration, myelination and sciatic nerve functional recovery in mouse sciatic nerve transection with a 3 mm gap, and in rat sciatic nerve transection with a 15 or 12 mm gap (Verdú et al., 1999; You et al., 2011; Zhang et al., 2019), and 20 mm gap (Guérout et al., 2011; Boecker et al., 2018), and with microsurgical nerve repair (Radtke et al., 2009a) and in rat sciatic nerve crush lesion (Dombrowski et al., 2006), as well as in rat facial nerve transection with a 5 mm gap (Guntinas-Lichius et al., 2001). As well, transplantation of olfactory mucosa significantly increases the accuracy of target reinnervation and accelerates the vibrissae movements (Guntinas-Lichius et al., 2002). Notably, olfactory ensheathing cell and Schwann cell, or and epidermal neural crest stem cell co-transplantation effectively enhance anatomical and functional repair after sciatic nerve injury in rats (You et al., 2011; Zhang et al., 2019) through enhancing the level of brain derived factor and nerve growth factor, which indicates that cells co-transplantation may serve as a new method for PNI in future therapies.

## Diabetic Neuropathy

Diabetic neuropathy is one of the most common complications of diabetic patients. With the incidences of diabetes increasing annually, especially in type 2 diabetes, studies are focused on understanding the pathogenic mechanisms, most of which are associated with neurons and vessels (Kim et al., 2012). However, accumulating evidence indicates the effect on morphological alterations and dysfunction in Schwann cells following diabetic neuropathy (Mizisin, 2014; Naruse, 2019). Studies on the sural nerves of rodents, cats and patients with diabetic neuropathy indicate that an apparently normal axon is wrapped by an abnormal myelin sheath resulting from segmental demyelination and remyelination (Thomas and Lascelles, 1965; Sima et al., 1988; Malik et al., 2005; Lennertz et al., 2011). In addition, the ultrastructure of abnormal Schwann cells showed mitochondrial enlargement with numerous vacuoles, cytoplasmic expansion, glycogen inclusion, and hyperplasia of the basement membrane (Yagihashi and Matsunaga, 1979; Chowdhury et al., 2013; Mizisin, 2014). Metabolic and molecular perturbations of Schwann cells in diabetic neuropathy include high activity of aldose reductase-mediated polyol pathway flux, oxidative stress and inflammation, as well as damage associated with microvascular changes in Schwann cells, all of which result in decreased neurotrophic factors release and the accumulation of neurotoxic intermediates leading to the dysfunction of interactions between Schwann cells and axons and diabetic neuropathy (Gonçalves et al., 2017, 2018; Naruse, 2019). Therefore, treating Schwann cells offers a potential strategy for diabetic neuropathy. Here, we primarily review the role of Schwann cells in cell therapy for diabetic neuropathy.

As shown in Table 3, different sources of stem cells exhibit potential of treating diabetic neuropathy and have an effect on the function of Schwann cells. BM-derived cells, endothelial progenitor cells (EPCs), and mononuclear cells (MNCs) can effectively reverse the symptoms of diabetic neuropathy through neuroprotective effects and neovascularization (Naruse et al., 2005; Hasegawa et al., 2006; Jeong et al., 2009; Kim et al., 2009). During this process, these neurotrophic and angiogenic factors suppress Schwann cell apoptosis and enhance Schwann cell proliferation and myelination (Jeong et al., 2009; Kim et al., 2012). In addition, treatment with BMSCs in hindlimb muscles, which can be differentiated into Schwann cell-like cells (Caddick et al., 2006), can ameliorate diabetic neuropathy symptoms, such as dysfunction of sensory and motor nerves, as well as demyelination in streptozotocin (STZ)-induced diabetic rats (Han et al., 2016). In addition to BMSCs, ASCs were transplanted by intramuscular injection and had a positive effect on the repair of STZ-induced diabetic neuropathy through the regulation of Schwann cell-related neurotrophic factor expression and remyelination (Yigitturk et al., 2021). Compared with stem cell-based treatment for diabetic neuropathy, additional studies have been performed with dental pulp stem cells (DPSCs). After human DPSCs were injected into the hindlimb skeletal muscle of diabetic mice, increases in vascular endothelial growth factor and nerve growth factor were detected at the injection site, while antibody neutralization reversed the effect of human DPSCs (Hata et al., 2020). Moreover, in STZ-induced diabetic rats, rat DPSCs ameliorated long-term (52 weeks) diabetic neuropathy (Omi et al., 2017). Although GFP-labeled rat DPSCs did not differentiate into Schwann cells after being injected into skeletal muscles (Hata et al., 2015), they had a beneficial effect on Schwann cells, including increasing Schwann cell viability and myelin formation (Omi et al., 2017). Of note, there was no difference in the therapeutic effect on diabetic neuropathy between the injection of rat DPSC-secreted factors and DPSCs (Kanada et al., 2020), and DPSC-secreted factors promoted Schwann cell proliferation and myelin formation (Omi et al., 2017). Conditioned medium from ASCs was also beneficial in preventing foot ulcer formation, ameliorating diabetic neuropathy in diabetic BKS *db/db* mice, and blocking diabetes-induced Schwann cell apoptosis (De Gregorio et al., 2020). Human DPSCs were used to treat a rat model of diabetic neuropathy through intramuscular or intravenous administration of one or two rounds of transplantation were helpful in contributing to functional recovery, but repeated doses *via* the intramuscular route was the most effective (Datta et al., 2017), which indicates that different routes and doses produce different effects. Neural crest can differentiate into multiple types of cells, including Schwann cells and peripheral neurons (Bronner and LeDouarin, 2012). However, there has only been one study using neural crest-like cells derived from induced pluripotent stem cells to treat STZ-induced diabetic mice, and the transplanted cells differentiated into Schwann cell-like cells or vascular smooth muscle cells to effectively improve the impaired vascular and neuronal functions (Okawa et al., 2013). Although the effect of Schwann cells as a cell therapy needs further study, Schwann cells are a key player in the treatment of diabetic neuropathy through cell transplantation.

**TABLE 3:** The effect of different cell therapies on Schwann cells for diabetic neuropathy.

Stem cell type	Cell source	Effect on Schwann cells	Notes
MNCs	Bone marrow/peripheral blood	Increased angiogenic and neurotrophic factor release (Hasegawa et al., 2006; Kim et al., 2009)	• Implantation into hindlimb muscles in STZ-induced diabetic rats; • Improvement in vascularity and motor nerve conduction velocity
EPCs	Bone marrow (Jeong et al., 2009)/ cord blood (Naruse et al., 2005)	• Decreased Schwann cells apoptosis and enhanced proliferation (Naruse et al., 2005; Jeong et al., 2009)	• Cell were injected into the hindlimb of STZ-induced diabetic mice or rats; • Enhancement in neural neovascularization and neuroprotective effects
BMSCs	Bone marrow	• Differentiation into Schwann cell-like cells and the upregulation of neurotrophic factors and myelination-related genes (Han et al., 2016)	• Injection into the hindlimb muscles of STZ-induced diabetic rats; • Increases in angiogenesis, neural function and myelination
ASCs	Adipose tissue	• Effects on the Schwann cell signal network, including neurotrophic effects and the restoration of myelination (Yigitturk et al., 2021)	• Injection into the thigh and lower hind-leg muscles of STZ-induced diabetic mice; • Restoration of neural structure and function
		• Reduced Schwann cell apoptosis with ASCs-conditioned medium (De Gregorio et al., 2020)	• Systemic administration in diabetic BKS *db/db* mice; • Avoiding foot ulcer formation and ameliorating polyneuropathy
DPSCs	Teeth	• Increased viability and myelin-related protein expression in Schwann cells (Omi et al., 2017) • Promotion of Schwann cells proliferation and myelin formation (Omi et al., 2017)	• Transplantation of human DPSCs into the hindlimb skeletal muscles of STZ-induced diabetic nude mice; • Treatment of diabetic polyneuropathy *via* the angiogenic and neurotrophic mechanism of hDPSC-secreted factors (Hata et al., 2020)
			• Transplantation of rat DPSCs into the hindlimb skeletal muscles of STZ-induced diabetic rats; • Improvements in long-term diabetic polyneuropathy (Omi et al., 2017)
			• Transplantation of freshly isolated and cryopreserved rat DPSCs into the hindlimb skeletal muscles of STZ-induced diabetic rats; • Amelioration of diabetic polyneuropathy (Hata et al., 2015)
			• Transplantation of rat DPSCs or administration of secreted factors into the hindlimb skeletal muscles of STZ-induced diabetic rats; • Amelioration of diabetic polyneuropathy with either treatment (Kanada et al., 2020)
			• Transplantation into STZ-induced neuropathic rats through the intramuscular or intravenous route *via* a single or two repeat doses; • Contribution to functional recovery with all treatments, but repeated doses *via* the intramuscular route was the most effective (Datta et al., 2017)
Neural crest cells	Induced pluripotent stem cells	Differentiation into Schwann cell-like cells (Bronner and LeDouarin, 2012)	• Transplantation into the hindlimb skeletal muscles of STZ-diabetic mice; • Improvement in impaired vascular and neuronal functions (Okawa et al., 2013)

## Chemotherapy-Induced Peripheral Neuropathy

Chemotherapy-induced peripheral neuropathy (CIPN) is the most common secondary effect in cancer patients who receive chemotherapy treatment. Signs of damage to peripheral nerves in CIPN are associated with sensory abnormalities, including allodynia (loss of touch sensation, numbness) or hyperalgesia (pin sensation and tingling), and often manifest as glove-stocking distributions (Bobylev et al., 2015). Some patients also exhibit motor nerve damage and altered musculoskeletal adverse effects (Ibrahim and Ehrlich, 2020). With the increasing numbers of cancer survivors and no ways to predict who will develop symptoms or when, there are no effective approved drugs to prevent or reduce CIPN (Carozzi et al., 2015; Jordan et al., 2019). Therefore, the management of CIPN is still a major challenge for clinical treatment. Despite the lack of direct evidence to illustrate the role of Schwann cell transplantation in CIPN, more attention has been given to the impairment of Schwann cells by chemotherapeutic agents and stem cell therapy for CIPN (Table 4; Al-Massri et al., 2020; Ibrahim and Ehrlich, 2020).

**TABLE 4:** The effect of chemotherapy on Schwann cells.

Anticancer agents	Symptoms	Effect on Schwann cells	Notes
Bortezomib	Severe sensory ataxia	Myelin damage (Filosto et al., 2007) Acute and transient endoplasmic reticulum damage to Schwann cells, abnormal myelination of Remak bundles and downregulation of myelin-related genes (Shin et al., 2010)	*In vitro* and *in vivo* experiments demonstrated the side effect of bortezomib on Schwann cells (Filosto et al., 2007; Shin et al., 2010)
Oxaliplatin, cisplatin, paclitaxel	Numbness, dysesthesia, paresthesia and muscle weakness	Disruption of myelin formation and mitochondrial dysfunction in Schwann cells	The cytotoxicity-induced by these drugs requires a lower dose in Schwann cells than in the dorsal root ganglion; The effect of these drugs on Schwann cells are different (Imai et al., 2017)
Epirubicin/docetaxel	Pain	Improvements in Schwann cell dedifferentiation	The side effect was suppressed by concomitant treatment with duloxetine and allopregnanolone (Matta et al., 2020)

Bortezomib, a proteasome inhibitor, is widely used in the treatment of multiple myeloma and induces axonal-dependent sensory damage and pathological responses in Schwann cells. Bortezomib-treated Schwann cells were analyzed by gene expression microarray, and the results indicated endoplasmic reticulum damage to Schwann cells accompanied by the downregulation of myelin-related genes, which was verified in a patient with high-dose bortezomib-induced peripheral neuropathy (Filosto et al., 2007; Shin et al., 2010). In contrast, compared with that in dorsal rooting ganglion neurons, a lower dose of oxaliplatin, cisplatin or paclitaxel is required in cultured Schwann cells because of cytotoxicity, and these drugs have a negative effect on myelin formation in cocultures but do not affect neurons, which indicates that Schwann cells are more susceptible to CIPN than other cells. Surprisingly, mitochondrial dysfunction occurs in cisplatin- and oxaliplatin-treated Schwann cells but not in paclitaxel-treated Schwann cells, while only paclitaxel induces Schwann cell dedifferentiation (Imai et al., 2017). Consistently, Schwann cell dedifferentiation occurs in epirubicin-docetaxel-induced CIPN, and this effect is suppressed by concomitant duloxetine-allopregnanolone treatment (Matta et al., 2020).

Mesenchymal stem/stromal cell therapy, which is a potential strategy for CIPN treatment, has a beneficial effect on improving symptoms (Al-Massri et al., 2020). MSC treatment could protect both sensory and motor neurons and enhance the efficacy of pregabalin in paclitaxel-induced peripheral neuropathy (Al-Massri et al., 2019). Notably, nasal administration of MSC-based therapy reverses cisplatin- or paclitaxel-induced peripheral neuropathy by Boukelmoune et al. (2021). In addition, ASCs also have a positive role in alleviating oxaliplatin-induced peripheral neuropathy (Di Cesare Mannelli et al., 2018). Induced pluripotent stem cells (iPSCs) can serve as a new method to estimate the neurotoxicity associated with chemotherapy treatment (Wheeler et al., 2015; Wing et al., 2017). The mechanisms of MSC-based therapies, including whether MSCs can differentiate into Schwann-like cells, need further study. MSC-based cell therapy may be a promising strategy for patients suffering from the adverse effects of cancer treatment.

## Conclusion and Future Perspectives

Emerging evidence has demonstrated the important role of Schwann cell/Schwann cell-like cell therapy in alleviating peripheral neuropathy, but a variety of challenges still need to be investigated. The source of both autological and allogenic Schwann cells are primarily nerve biopsies and traumatized nerve stumps, all of which will result in the innervation of anatomical regions for the donor and undesired morbidities. Moreover, nerve-derived Schwann cells need a long expansion time *in vitro* to produce a large number of cells. The time between injury and transplantation with Schwann cells should be minimized to protect patients from a series of secondary injuries, including muscle degeneration and functional loss. Given these limitations, Schwann cell-like cells from stem cells have become a relatively robust alternative cell for the repair of peripheral neuropathy. The therapeutic application of pluripotent stem cells is associated with safety and technical and ethical constraints compared with other stem cell types, such as BM-MSCs, dM-MSCs and SKPs. However, these cells have a long differentiation time after isolation and can delay treatment, resulting in further damage to the patient. Highly efficient methods for *in vitro* differentiation and characterization of Schwann cell-like cells may support future clinical applications. On the other hand, direct transplantation with these stem cells, followed by *in vivo* differentiation associated with the pathological stage of peripheral neuropathy, may become a promising and attractive therapeutic strategy. In addition, in patients transplanted with allogenic Schwann cells or Schwann cell-like cells, drugs still need to be used to avoid immune rejection and potential side effects.

Although there is a large amount of evidence on the role of Schwann cell-like cells in peripheral neuropathy in rodent animal models, including peripheral nerve injury, diabetes and chemotherapy, until now, no direct clinical trials have been developed with these cells. However, for spinal cord injury, several studies reported the therapeutic benefits of treatment with these cells. Most studies have been focused on evaluating safety and adverse events after transplantation (Yazdani et al., 2013; Mendonca et al., 2014; Anderson et al., 2017; Gant et al., 2021). Notably, single MSC administration is safe but less effective than combination treatment with autologous Schwann cells (Oh et al., 2016), which improves sensory and motor functional recovery to some extent, as well as bladder compliance (Oraee-Yazdani et al., 2016, 2021). In addition, the administration of these cells by intravenous infusion, intrathecal administration or direct injection into spinal lesions, and the injury level and size may lead to different outcomes. Due to the effect of advanced age (Tong et al., 2015; Liu et al., 2018) and sexual dimorphism (Magnaghi et al., 2006; Stenberg and Dahlin, 2014) on the characterization of Schwann cells, these factors need to be taken into consideration when choosing a therapeutic strategy. Therefore, many more preclinical studies with cell therapies are needed prior to clinical application.

Schwann cells release a variety of signaling molecules under both physiological and pathological conditions to promote neuronal development and postinjury regeneration (Monje, 2020). Therefore, whether appropriate signaling molecules or drugs are administered in combination with transplanted Schwann cells still needs to be carefully assessed (Balakrishnan et al., 2020). In addition to the variability in the repair response between rodent and human models, relatively long-distance transection or injury occurs in humans compared with rodent models, and a slow rate of nerve regeneration requires a longer time for transplanted Schwann cells or Schwann cell-like cells to support and remyelinate the regenerated axons (Balakrishnan et al., 2020).

In summary, although a great number of challenges remain to be addressed, a growing body of evidence demonstrates the beneficial therapeutic roles of Schwann cells and Schwann cell-like cells in peripheral neuropathy. With deeper insights into the pathology of peripheral neuropathy-related disorders, including peripheral nerve injury, diabetes and chemotherapy, as well as the development of bioengineering systems, Schwann cell-based therapy will soon be a more attractive and effective strategy for treating peripheral neuropathy.

## Author Contributions

Z-YW and GC conceptualized the topics. Z-YW, QW, Z-ML, F-YC, W-FS, and Y-YZ wrote and revised the manuscript. All authors contributed to the article and approved the submitted version.

## Conflict of Interest

The authors declare that the research was conducted in the absence of any commercial or financial relationships that could be construed as a potential conflict of interest.

## Publisher’s Note

All claims expressed in this article are solely those of the authors and do not necessarily represent those of their affiliated organizations, or those of the publisher, the editors and the reviewers. Any product that may be evaluated in this article, or claim that may be made by its manufacturer, is not guaranteed or endorsed by the publisher.
